# Supplementary material for: Dysphagia Care and Management in Rehabilitation: A National Survey
Source: J Clin Med. 2022 Sep 27;11(19):5730. doi: 10.3390/jcm11195730 (PMC9571108; doi:10.3390/jcm11195730)
Supplement: Supplementary file 1 [file jcm-11-05730-s001.zip › jcm-1916987-supplementary.pdf]

Dysphagia care and management in rehabilitation: a national survey

Renée Speyer[1,2,3], Adriana Sandbekkbråten[1,4], Ingvild Rosseland[5], Jennifer L. Moore[6,7]

[1]Department Special Needs Education, University of Oslo, 0318 Oslo, Norway

[2]Curtin School of Allied Health, Faculty of Health Sciences, Curtin University, Perth, WA 6102, Australia

[3]Department of Otorhinolaryngology and Head and Neck Surgery, Leiden University Medical Centre, 2333 ZA Leiden, The Netherlands

[4]Aurskog-Høland municipality, 1940 Bjørkelangen, 1940 Viken, Norway

[5]Reinforced Interdisciplinary Rehabilitation Aker, City of Oslo, 0586 Oslo, Norway.

[6]Institute for Knowledge Translation, Carmel, IN, 46033, USA

[7]Regional Center of Knowledge Translation in Rehabilitation, Aker hospital, 0586 Oslo, Norway

Supplementary file S1: NATIONAL SURVEY IN HEALTH HOUSES AND REHABILITATION CENTRES[a,b]

START (All respondents: managers and healthcare professionals)

Participants

1.1 Are you an employee at a health house or a rehabilitation centre?

1.2 What is your position at the health house or rehabilitation centre where you work (e.g., director of care, executive officer, department manager, head of allied health, nurse, occupational therapist, speech therapist)?

1.3 What is your educational / professional background (e.g., health management, medical doctor, nurse, physiotherapist, occupational therapist, speech therapist)?

1.4 What are your main responsibilities within the health house of rehabilitation centre (i.e., managerial activities or clinical activities)?

I SURVEY FOR MANAGERS

Rehabilitation centres and health houses

2.1 How many beds are available at the health house or rehabilitation centre?

2.2 What type of care is provided at your health house or rehabilitation centre (e.g., day-care, inpatient short-term stay, inpatient long-term stay, outpatient care, home healthcare)?

2.3 Which diagnostic groups of clients are eligible to use your services (e.g., dementia, neurodegenerative diseases, traumatic brain injury, stroke, head and neck or oesophageal cancer, congenital neurological conditions)?

2.4 Which professionals are working in your health house or rehabilitation centre (e.g., manager, medical doctor, psychologist, nurse, physiotherapist, occupational therapist, speech therapist, social worker, nutritionist, care assistants, personnel without a professional degree)?

Of the professionals that you listed above, how many fulltime equivalent staff do you have?

Screening and assessment for dysphagia

3.1 How high would you estimate the percentage of clients (per diagnostic group) that have eating and swallowing difficulties? E.g., dementia, neurodegenerative diseases, traumatic brain injury, stroke, head and neck or oesophageal cancer, and congenital neurological conditions.

3.2 How are clients screened for eating and swallowing difficulties (e.g., client's self-report or caregiver's report, mealtime observation, screening for eating and swallowing difficulties, clinical assessment for eating and swallowing difficulties, report by external healthcare professionals, screening for malnutrition and dehydration)?

3.3* When are clients screened for eating and swallowing difficulties (e.g., screening before arrival at health house or rehabilitation centre, screening at arrival at health house or rehabilitation centre, when changes in client's eating and swallowing are observed, when changes in client's cognitive and/or physical functioning are observed, once per week [weekly routine], once per month [monthly routine], no standard routine)?

3.4* Who usually screens or assesses clients for eating and swallowing difficulties (e.g., nurse, physiotherapist, occupational therapist, speech therapist, social worker, nutritionist, care assistants, personnel without a professional degree)?

3.6 What type of assessment is conducted routinely with clients at your health house or rehabilitation centre (e.g., swallowing function, dental status, need to adjust the consistency of food and drink, need to adjust medicine intake [e.g., change of consistency, crushed tablets], nutritional status)?

Dysphagia management and clinical practice in rehabilitation

4.1* What challenges or additional difficulties do clients with eating and swallowing difficulties experience (e.g., problems in communication, self-feeding or eye-hand coordination, drooling, problems with chewing caused by dental status, reduced appetite, weight loss or malnutrition, dehydration, coughing during or after eating or drinking, food residues in mouth after swallowing, changes or wet voice after drinking, problems with medicine intake, pneumonia)?

Dysphagia training and education

5.1 Do you have required educational training for staff in eating and swallowing difficulties at your health house or rehabilitation centre?

5.2* Which opportunities for professional development in eating and swallowing difficulties are available to staff (e.g., theoretical upskilling [e.g., webinars, internal courses], onsite training [e.g., workshop], coaching [by experienced staff or colleague], external expert course on eating and swallowing difficulties)?

5.3* What is the rehabilitation service's routine for upskilling kitchen staff responsible for preparing meals for clients with eating and swallowing difficulties (e.g., theoretical upskilling [e.g., webinars, internal courses], onsite training [e.g., workshop], external expert course on eating and swallowing difficulties)

Self-perceived quality of dysphagia care

6.1* How would you rate the quality of care for people with eating and swallowing difficulties at your health house or rehabilitation centre?

II SURVEY FOR HEALTHCARE PROFESSIONALS

Screening and assessment for dysphagia

2.1 Who decides which clients will be screened for being at risk of eating and swallowing difficulties? E.g., all clients are screened, medical doctor decides who needs to be screened, client's referral includes screening recommendation.

2.2 Do you screen clients for being at risk of eating and swallowing difficulties?

2.3 Which screening tools do you use and how often?

2.4* When are clients screened for eating and swallowing difficulties (e.g., screening before arrival at health house or rehabilitation centre, screening at arrival at health house or rehabilitation centre, when changes in client's eating and swallowing are observed, when changes in client's cognitive and/or physical functioning are observed, once per week [weekly routine], once per month [monthly routine], no standard routine)?

2.5 Do you assess clients for being at risk of eating and swallowing difficulties?

2.6 Which a assessments do you use and how often?

2.7 When are clients assessed for eating and swallowing difficulties (e.g., assessment before arrival at health house or rehabilitation centre, assessment at arrival at health house or rehabilitation centre, when changes in client's eating and swallowing are observed, when changes in client's cognitive and/or physical functioning are observed, once per week [weekly routine], once per month [monthly routine], no standard routine)?

2.8* Who usually screens or assesses clients for eating and swallowing difficulties (e.g., nurse, physiotherapist, occupational therapist, speech therapist, social worker, nutritionist, care assistants, personnel without a professional degree)?

Dysphagia management and clinical practice in rehabilitation

3.1* What challenges or additional difficulties do clients with eating and swallowing difficulties experience (e.g., problems in communication, self-feeding or eye-hand coordination, drooling, problems with chewing caused by dental status, reduced appetite, weight loss or malnutrition, dehydration, coughing during or after eating or drinking, food residues in mouth after swallowing, changes or wet voice after drinking, problems with medicine intake, pneumonia)?

3.2 Which strategies and routines do you use to support clients with eating and swallowing difficulties? E.g., improving clients' upright sitting posture, adjusting head positioning, use of customized mealtime utensils, modification of food consistencies, modification of liquid consistencies, change of medicine intake (e.g., change of consistence, crushed tablets), changes in environment (e.g., avoidance of distracting background activities or noise [e.g., television, music]), mealtime observation, checking for clients to be well rested and alert during mealtimes, offering hand support during eating, control of bolus size per bite or sip, checking for food residues in mouth, controlling speed of oral intake, having clients actively engaged in drinking and eating activities, allowing prolonged upright sitting after mealtimes for at least 15 minutes, oral care after meals?

3.3 Which treatment techniques do you use when treating clients with eating and swallowing difficulties? E.g., oral motor exercises, super supraglottic manoeuvre and supraglottic manoeuvre, Mendelsohn manoeuvre, Shaker-exercise, Effortful swallow, Masako manoeuvre, other swallowing manoeuvres, chin tuck, head tilt, head rotation, other changes in head positioning, thermal tactile stimulation, neuromuscular electrical stimulation (NMES).

3.4 What would be the average period that clients are being treated for eating and swallowing difficulties?

3.5 What would be the average number of session that clients receive when being treated for eating and swallowing difficulties?

3.6 What would be the average duration of a session that clients receive when being treated for eating and swallowing difficulties?

3.7 Are clients with eating and swallowing difficulties being treated individually or in groups?

3.8 Do you have access to external clinical professionals for assessment and treatment of clients with eating and swallowing difficulties?

3.9 Who is responsible for preparing meals for clients with eating and swallowing difficulties?
E.g., external kitchen facilities, health house or rehabilitation kitchen staff, clients' relatives?

3.10 Which classification system do you use for clients who need adapted consistency of food, drink and or medicine. E.g., dietary handbooks, International dysphagia diet standardisation initiative (IDDSI), classification system developed by health house or rehabilitation staff)?

Dysphagia training and education

4.1 Do you attend required educational training in eating and swallowing difficulties at your health house or rehabilitation centre?

4.2* Which opportunities for professional development in eating and swallowing difficulties are available to you (e.g., theoretical upskilling [e.g., webinars, internal courses], onsite training [e.g., workshop], coaching [by experienced staff or colleague], external expert course on eating and swallowing difficulties)?

4.3* What is the rehabilitation service's routine for upskilling kitchen staff responsible for preparing meals for clients with eating and swallowing difficulties (e.g., theoretical upskilling [e.g., webinars, internal courses], onsite training [e.g., workshop], external expert course on eating and swallowing difficulties)

Self-perceived quality of dysphagia care

5.1* How would you rate the quality of care for people with eating and swallowing difficulties at your health house or rehabilitation centre?

[a] Original survey developed in Norwegian.

[b] Survey headings refer to corresponding paragraphs in the results section of the article.

* Overlap between survey for managers and survey for healthcare professionals.

Note. The survey for managers consisted of 7 multiple choice questions, 5 matrix questions, 2 numeric textbox questions, and one ordinal scale question. The survey for healthcare professionals consisted of 13 multiple choice questions, 6 matrix questions, 2 ordinal scale questions, and 2 open-ended questions. The survey contained short explanations where appropriate on topics such as 'dysphagia' or 'screening and assessment'. Throughout both surveys, participants could elaborate on questions using open comment boxes.
